# Adenocarcinoma of the Breast Presenting as Occult Breast Cancer With Axillary and Supraclavicular Lymph Node Metastasis: A Case Report

**DOI:** 10.7759/cureus.39583

**Published:** 2023-05-27

**Authors:** Andy Aleman Espino, Isabel C Bernal, Jesus E Guarecuco, Rana El-Tawil, Mohammed M Masri

**Affiliations:** 1 Medicine, Nova Southeastern University Dr. Kiran C. Patel College of Osteopathic Medicine, Fort Lauderdale, USA; 2 General Surgery, Larkin Community Hospital, South Miami, USA

**Keywords:** lymph node metastasis, breast cancer, adenocarcinoma, modified radical mastectomy, modified radical mastectomy (mrm), lymphadenopathy, occult breast cancer, case report

## Abstract

Breast cancer is a leading cause of cancer-related mortality in women, with over 250,000 new cases diagnosed annually in the United States. Although mortality rates have decreased, breast cancer remains the second most common cause of cancer death in women. Occult breast cancer (OBC), a rare form of breath cancer that typically presents as axillary lymphadenopathy with no evidence of primary disease, accounts for less than 1% of all breath cancer diagnoses. To date, only three cases of OBC treated with radical mastectomy have been documented in the literature. This case presents a 76-year-old female with a benign left breast mass who was subsequently diagnosed with metastatic estrogen receptor/progesterone receptor (ER/PR)-positive ductal cell breast carcinoma after a visible axillary lymph node was detected on follow-up imaging. Due to the rarity of OBC, standardized treatment guidelines have not been established. Our patient underwent a left radical mastectomy with axillary and cervical lymph node dissection. Clinicians should maintain a high index of suspicion for biopsying axillary lymph nodes in females without evidence of breast malignancy, even though OBC has a low incidence rate. This case report aims to present a documented case of OBC and comprehensibly review the existing literature, discussing the available diagnostic and treatment approaches for this condition.

We describe the case of a 76-year-old woman referred to surgery consultation due to a mammographic finding of a left superior lateral mass. The mass was biopsied and found to have no malignancy. On follow-up imaging, she was found to have a left axillary lymph node visible. Her only complaints at this time were breast tenderness and swelling. She underwent fine needle aspiration of the mass, which showed atypical cells that led to an excisional biopsy of the detected axillary node. The biopsy pathology report showed ER/PR-positive ductal cell breast carcinoma. The patient underwent left modified radical mastectomy with left axillary and cervical lymph node dissection. It was during this procedure that the pathology report revealed a 2 cm lesion on the left breast that showed ER/PR-positive infiltrating ductal carcinoma with 32 out of the 37 lymph nodes positive for metastatic disease.

This case illustrates the importance of having a low imaging threshold in patients with vague breast symptoms. Surgeons should have a high level of suspicion when metastatic breast cancer is found, even if there is no clinical or radiographic evidence of a primary lesion. This includes conducting lymph node biopsies in patients who present with lymphadenopathy without the initial presence of primary breast cancer. Many studies agree that a modified radical mastectomy with lymph node recession is the treatment of choice for metastatic breast cancer without evidence of primary lesion. However, the efficacy of adjuvant treatments like radiation therapy or chemotherapy should be further studied.

## Introduction

Over 250,000 women in the United States are diagnosed with breast cancer yearly, and although mortality has declined, it is still the second most common cause of cancer death in women [1]. Data from the Surveillance, Epidemiology, and End Results (SEER) program show infiltrating ductal carcinoma as being the most reported (76%), followed by invasive lobular (8%) and ductal/lobular (7%) [2]. Invasive breast carcinoma has many different histological origins and molecular subtypes. Determining the type of invasive carcinoma becomes vital to understand the response to treatment and outcomes. Molecular subtypes are determined using biological markers, including estrogen (ER) or progesterone (PR) receptors, levels of human epidermal growth factor receptor 2 (HER2) protein, and the number of copies of the HER2 gene [3]. Ki67 indicates large amounts of actively dividing cells correlated to this subtype's high-grade tendencies and poor survival rates [4]. Triple-negative cancers are more common in black women and have the worst prognosis because, currently, there are no targeted therapies for this tumor [5].

Occult breast cancer (OBC) refers to the presence of a metastatic breast malignancy whose primary origin is unknown [6]. Although breast cancer is one of the most common cancers in the female population, carcinoma of unknown primary (CUP) accounts only for a small percentage of these. The OBC incidence is 0.1-0.8% of new breast cancer diagnoses [7]. The most common presentation of OBC is axillary lymphadenopathy. Although palpable/enlarged axillary lymph nodes are generally related to benign disease, a biopsy should be performed to rule out the possibility of malignancy [8]. The standard treatment for OBC is to perform a modified radical mastectomy with axillary lymph node dissection [9]. The role of breast conservation or radiation therapy is still unclear. More studies should be performed to assess its efficacy compared to standard treatment [10]. The presence of markers like ER and PR are good prognostic factors that will open the possibility for endocrine therapy as an adjuvant treatment once the surgery is performed [11]. This report aims to review the available literature and discuss the diagnosis and treatments available for OBC.

## Case presentation

**Figure 1:**
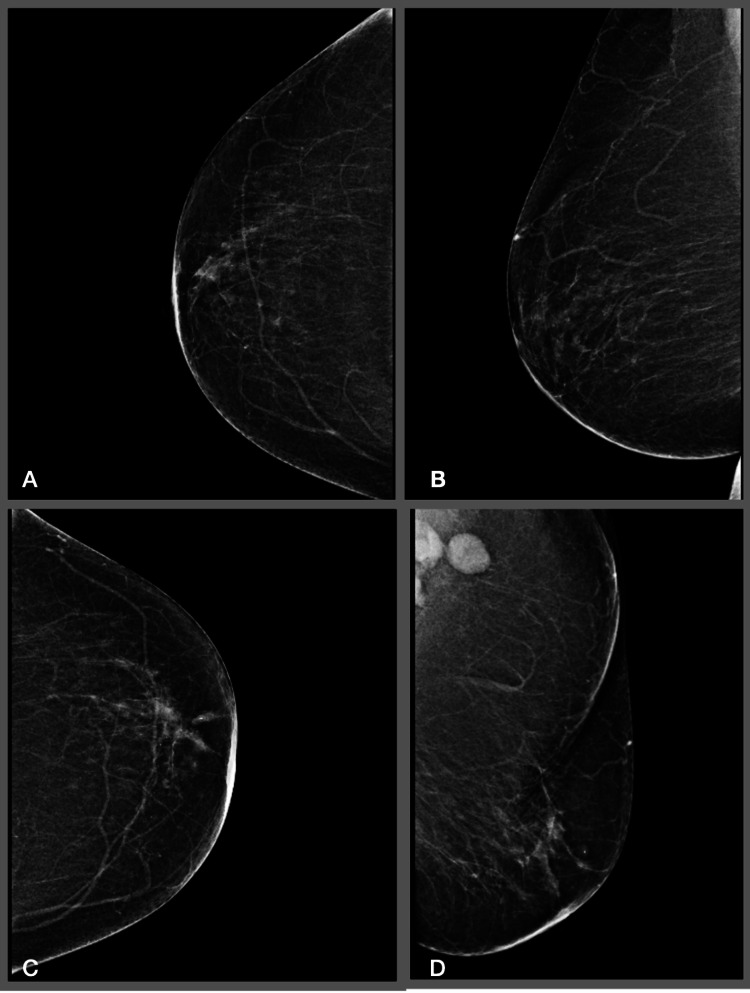
Mammogram remarkable for left axillary lymph node mass with surrounding fatty hilum (D). A: Right craniocaudal view (R-CC). B: Right mediolateral oblique view (R-MLO). C: Left craniocaudal view (L-CC). D: Left mediolateral oblique view (L-MLO).

**Figure 2:**
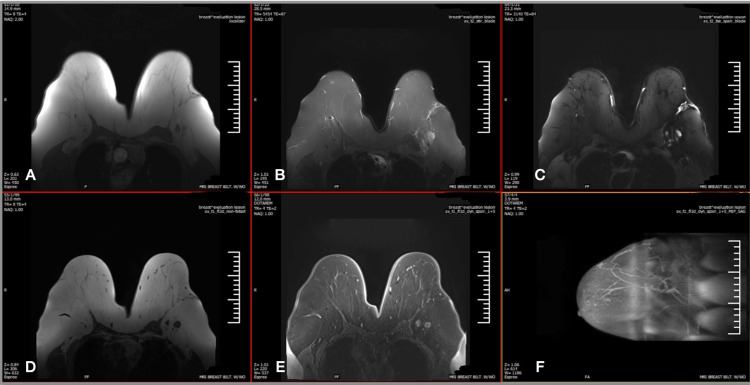
MRI of the breasts. A: Breast evaluation lesion localizer. B: T2 short tau inversion recovery (STIR). C: T2 spectral attenuated inversion recovery (SPAIR). D: T1 fast low-angle shot three-dimensional imaging (FL3D). E: T1 SPAIR. F: T1 SPAIR maximum intensity projection (MIP) sagittal.

A 76-year-old post-menopausal woman G1P1 with a past medical history of hypertension, diabetes mellitus, deep vein thrombosis (DVT), and mesenteric vein thrombosis presented for further examination due to a concerning routine mammogram finding that showed a lesion superior lateral to the left nipple measuring approximately 2 cm and showed a mass effect. At the time, the patient also complained of breast pain and itchiness. Physical exam was remarkable for a left tender mobile breast lump, dimpling of the surface of the breast, and retraction of the nipple. Ultrasound (US) studies confirmed the presence of this lesion. Fine needle aspiration (FNA) and core needle biopsy were significant for benign mammary tissue and negative for atypia or malignancy. On follow-up mammogram (Figure 1) and US, she had a left axillary lymph node mass of 2.3 cm with surrounding fatty hilum, though her only complaints were left breast tenderness and swelling. FNA of the axillary lymph node mass was significant for atypical cells, and a decision was made to proceed with a US-guided excisional biopsy of the suspicious left axillary lymph node. The results yielded ER/PR-positive ductal cell breast carcinoma. A bilateral breast MRI (Figure 2) demonstrated left breast axillary tail inflammatory pathological adenopathy and a left axillary region (level 3) conglomerate of pathological adenopathy.

**Figure 3:**
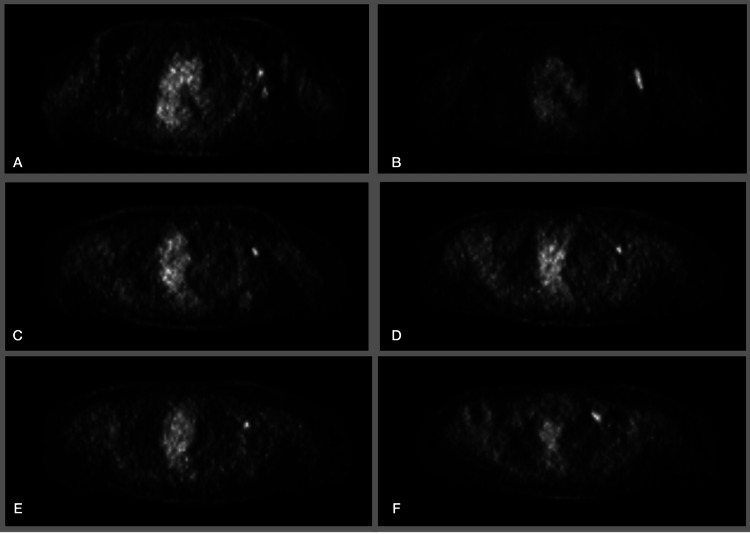
Positron emission tomography-computed tomography of the chest. A-E: Increased uptake in left axillary lymph nodes. F: Increased uptake in left supraclavicular lymph nodes.

**Figure 4:**
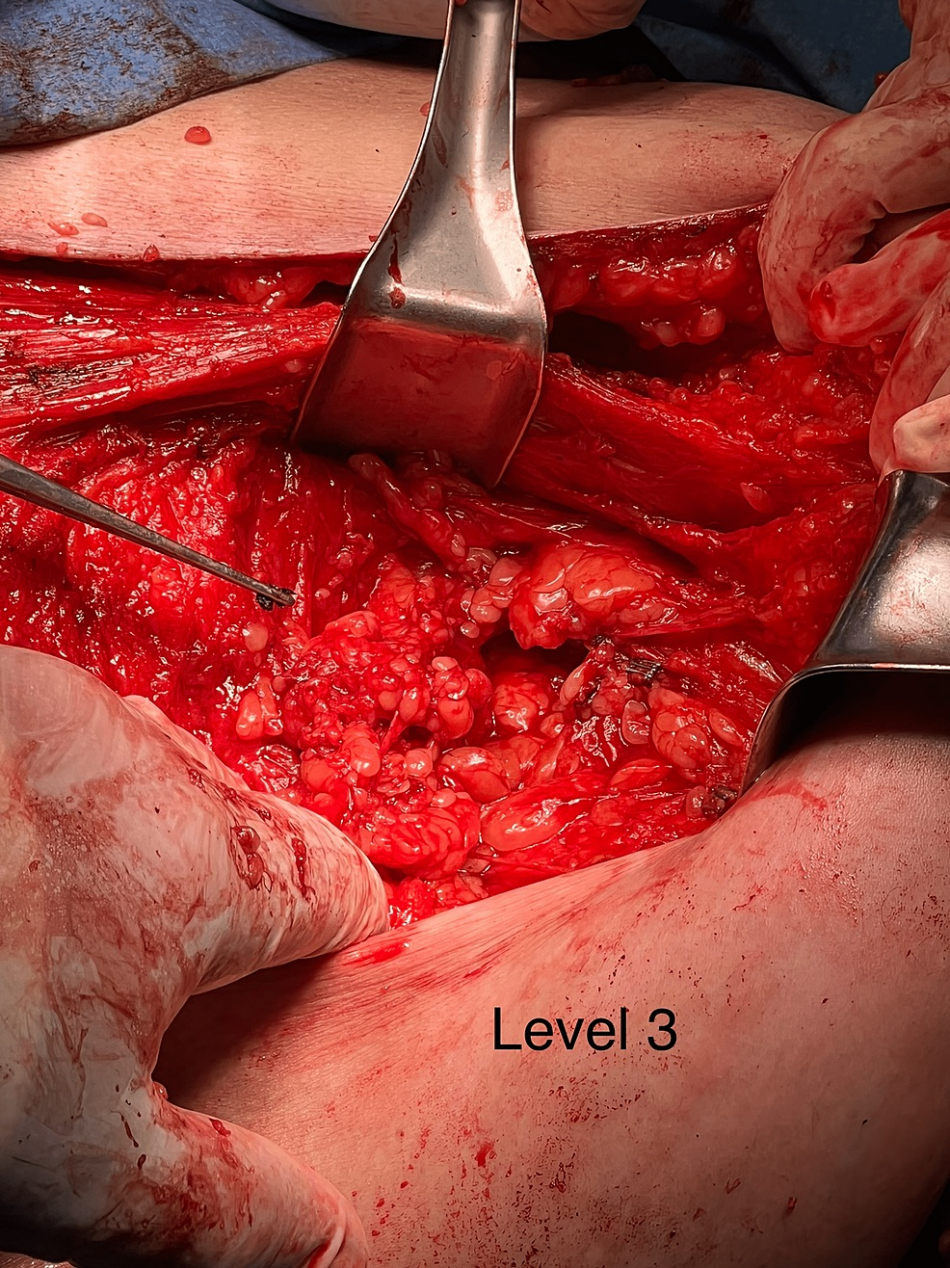
Photograph showing the dissection of left axillary level 3 lymph nodes.

Further workup with positron emission tomography-computed tomography (PET-CT) was done, which showed positive left supra clavicular lymph node and left axillary numerous lymph nodes (Figure 3). The patient was recommended to undergo left modified radical mastectomy with left axillary lymph node dissection with possible left cervical lymph node dissection. The patient agreed with the recommendation. The entirety of the breast tissue down to the pre-pectoral fascia of the serratus anterior muscle was removed, which included the left breast tail, and it was sent for pathology. Axillary lymph node dissection prompted the removal of level 1, 2, and 3 nodes (Figure 4). On further dissection, there were no concerning supraclavicular nodes identified. The dissection concluded with the removal of Rotter’s nodes and inferior cervical lymph nodes.

The pathology report noted a 2 cm lesion on the left breast that showed infiltrating ductal carcinoma histology with positive estrogen/progesterone receptor expression. In addition, 32 out of the 37 dissected lymph nodes were found to be positive for metastatic cancer, and thus the patient was referred to the oncology team. The patient was to receive four cycles of Adriamycin and cyclophosphamide and then weekly doses of paclitaxel for 12 weeks via a port. Then she would be referred to radiation therapy and would be placed on Verzenio to continue her treatment.

## Discussion

Breast cancer is the most common cause of axillary lymphadenopathy. Therefore, patients presenting with inflamed axillary lymph nodes without clinical evidence of a primary tumor should undergo a node biopsy for subsequent pathologic and immunohistochemical classification. A complete physical examination, mammography, and breast ultrasound should follow if the results confirm breast carcinoma. In case the results are negative, additional imaging, such as an MRI of the breasts, positron emission tomography (PET) scan, and nuclear medicine studies, should be conducted to locate the primary tumor [12]. If there is no radiological evidence of a primary tumor, cancer will be classified as CUP or OBC. In such cases, it is recommended to treat cancer according to stage II breast cancer guidelines, and axillary lymph node dissection (ALND) should be performed in all patients [13]. Radiotherapy (RT) may be used to treat the breast if breast conservation is desired, and mastectomy is the most recommended approach [14].

Currently, limited data support the benefits of RT as an alternative to mastectomy for breast conservation. A retrospective study by He et al. in 2012 followed 96 patients from 1998 to 2012, comparing patients who underwent ALND and mastectomy, ALND and RT, and ALND alone. The study concluded that patients receiving RT had similar outcomes to those who underwent a mastectomy. Although some other small retrospective studies also reported similar results, no randomized trials have compared the role of RT as an alternative treatment to radical mastectomy for OBC [13]. The efficacy of adjuvant systemic therapy has not been studied extensively. However, for ER/PR-positive tumors, adjuvant endocrine therapy is recommended since the effectiveness of aromatase inhibitors and estrogen modulators has been demonstrated in numerous randomized trials [13].

In this case, the patient presented with axillary lymphadenopathy without any evidence of breast masses or lumps upon physical examination. FNA of the lymph nodes revealed breast ductal cell carcinoma, but imaging failed to locate a primary tumor. PET scan results showed positivity in the left axillary and supraclavicular lymph nodes. This prompted the diagnosis of OBC, and a biopsy of the left axillary lymph nodes confirmed ductal cell carcinoma, which was ER/PR-positive. A left modified radical mastectomy with left ALND and possible neck node dissection was chosen as the treatment. During the pathological analysis of the surgical samples, a 2 cm mass was found within the breast tissue, changing the diagnosis of "occult breast cancer" since a primary mass was discovered during the pathological exploration. A study by Thompson et al. assessed the incidence of OBC in prophylactic mastectomies and found that the median malignancy ranged in size from 0.6 to 1.6 cm for the bilateral prophylactic mastectomy group and 0.1 to 3 cm for the contralateral mastectomy group [14]. The reason the 2 cm mass was not detected during breast ultrasound, MRI, or PET scan is unknown.

OBC is a rare type of breast cancer, representing only a small percentage of diagnosed breast cancers worldwide. It typically presents as axillary lymphadenopathy, as seen in this case, and in some cases, as supraclavicular lymphadenopathy. For this patient, the treatment of choice was ALND and mastectomy, with additional dissection of cervical lymph nodes accessed from the initial incision to avoid additional skin lacerations. The efficacy of other treatments like RT for breast conservation still requires further study in randomized control trials to yield significant results [13].

## Conclusions

We present a case where a 2 cm mass was identified during the pathological exploration of an excised left breast despite not being detected on ultrasound, MRI, and PET scans. The patient initially presented with axillary and supraclavicular lymph node metastasis, identified as ductal cell carcinoma. The treatment of choice was a modified radical mastectomy, with possible neck node dissection. This case illustrates the importance of having a low imaging threshold in patients with vague breast symptoms. Surgeons should maintain a high level of suspicion when metastatic breast cancer is detected, even in the absence of primary lesions. Many studies have demonstrated modified radical mastectomy with lymph node recession as the preferred treatment option. However, the efficacy of adjuvant treatments such as radiation therapy or chemotherapy warrants further study.
